# Improving random forest predictions in small datasets from two-phase sampling designs

**DOI:** 10.1186/s12911-021-01688-3

**Published:** 2021-11-22

**Authors:** Sunwoo Han, Brian D. Williamson, Youyi Fong

**Affiliations:** Vaccine and Infectious Disease Division, Fred Hutchinson Cancer Research Center, Seattle, USA

**Keywords:** Case–control design, Variable screening, Class imbalance, HIV vaccine

## Abstract

**Background:**

While random forests are one of the most successful machine learning methods, it is necessary to optimize their performance for use with datasets resulting from a two-phase sampling design with a small number of cases—a common situation in biomedical studies, which often have rare outcomes and covariates whose measurement is resource-intensive.

**Methods:**

Using an immunologic marker dataset from a phase III HIV vaccine efficacy trial, we seek to optimize random forest prediction performance using combinations of variable screening, class balancing, weighting, and hyperparameter tuning.

**Results:**

Our experiments show that while class balancing helps improve random forest prediction performance when variable screening is not applied, class balancing has a negative impact on performance in the presence of variable screening. The impact of the weighting similarly depends on whether variable screening is applied. Hyperparameter tuning is ineffective in situations with small sample sizes. We further show that random forests under-perform generalized linear models for some subsets of markers, and prediction performance on this dataset can be improved by stacking random forests and generalized linear models trained on different subsets of predictors, and that the extent of improvement depends critically on the dissimilarities between candidate learner predictions.

**Conclusion:**

In small datasets from two-phase sampling design, variable screening and inverse sampling probability weighting are important for achieving good prediction performance of random forests. In addition, stacking random forests and simple linear models can offer improvements over random forests.

**Supplementary Information:**

The online version contains supplementary material available at 10.1186/s12911-021-01688-3.

## Background

Prediction of a binary disease outcome from a collection of clinical covariates and biomarker measurements is a common task in biomedical studies. Many machine learning methods have been used with great success in solving problems as diverse as early prognosis and diagnosis of a cancer type [1], identifying rare disease [2], and prediction of infectious disease risk [3]. However, machine learning methods have not been widely adopted in the context of prevention clinical trials using two-phase sampling designs. Two-phase sampling [4] is a method to design substudies on selected subjects from a cohort to avoid measuring expensive covariates for every participant in the cohort. Typically, subjects in the cohort are classified into several strata based on the cohort information, and then a subset of subjects is randomly sampled without replacement from each stratum (see Additional file 1: Section D for a more detailed explanation.) Studies using the two-phase sampling designs often have a small number of disease endpoints and a high cost associated with measuring biomarkers such that only a small representative subset of controls have biomarker measurements. Most conventional machine learning methods tend to be unsuccessful in situations with small sample sizes because the methods require a substantial amount of training data.

Random forests [RF; 5] are a popular machine learning method that have been increasingly used in biomedical applications. For example, RF have been used to recognize cancer-associated biomarkers from clinical trial data [6], to predict protein-protein interactions [7, 8], and to identify informative genes for a disease from microarray gene expression data [9, 10]. RF has many advantages: it is fast in both model training and evaluation, is robust to outliers, can capture complex nonlinear associations, cope with class imbalance data, and produces competitive performance for high dimensional data [11, 12]. It has also been shown to handle challenges arising from small sample sizes [13]. In this manuscript, we seek to optimize random forest prediction performance using combinations of variable screening, class balancing, weighting, and hyperparameter tuning.

## Methods

We conduct our experiments on RF using an immunologic marker dataset from the HIV Vaccine Trials Network (HVTN) 505 trial, a phase III HIV preventative vaccine efficacy trial [14]. The trial contained a nested biomarker study to examine immunologic correlates of risk of infection using a two-phase sampling design, in which the vaccine recipients were stratified by body mass index (BMI) and race/ethnicity, five controls were randomly sampled from a stratum for each case therein, and an array of HIV-1-specific vaccine-induced T cell and antibody biomarkers were measured in 25 cases and in 125 controls [15–17].

It is of great interest to build a model that best predicts HIV infection risk from the set of immune response biomarkers and clinical covariates measured in the HVTN 505 trial. However, two potential challenges related to the small sample size can result in poor prediction performance. The first is that the immunologic marker dataset from the HVTN 505 study, which we will refer to as the HVTN 505 dataset, is high-dimensional: the total number of the biomarkers is 420, compared to the 150 observations. The second challenge is class imbalance, since the ratio of cases to controls is 1:5. In general, when the number of input variables is larger than the number of observations and the class distribution is skewed, the prediction performance of machine learning methods can deteriorate [11, 18].

We first give a brief introduction to RF, and then, we study variable screening, class balancing, and inverse sampling probability weighting. We also investigate the impact of hyperparameter tuning on the performance of RF. Furthermore, we compare the prediction performance of RF to that of generalized linear models (GLM) and propose several stacking models that combine the predictions of RF and GLM.

## Results

### Random forest optimization

#### Random forests

**Fig. 1:**
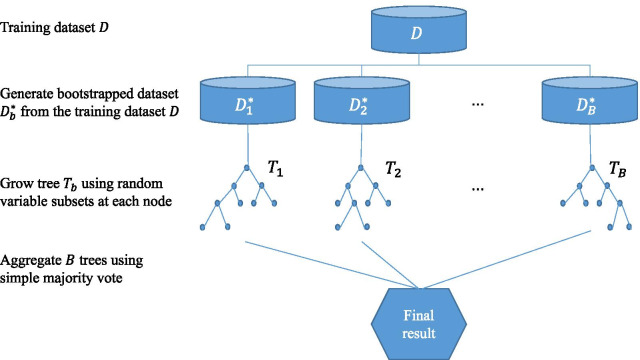
A flow diagram for the random forest algorithm in the context of classification

Random forests [RF; 5] are a popular classification and regression ensemble method. The algorithm works by building multiple individual classifiers (or regression functions) and then aggregating them to make a final prediction. The most widely used implementations of RF are tree-based ensembles consisting of classification and regression trees [CART; 19]; however, other methods can be applied as well. Random forests are trained by generating bootstrapped datasets from an initial training dataset; next, trees are fitted on the bootstrapped datasets to maximal depth without pruning [5, 20]. To construct each individual tree, the algorithm searches for the best split criterion on a random subset of the variables instead of all variables at each node. This randomness causes trees to be more diverse; as a result, aggregating multiple uncorrelated trees significantly reduces the variance of the estimator and improves overall performance. For prediction on a given test observation, the class predicted by each individual tree is aggregated to make a final prediction using a simple majority vote in classification problems. We present a flow diagram of the algorithm in the context of classification in Fig. 1.

#### Variable screening, class balancing, and inverse sampling probability weighting

Following a slightly simplified version of the analysis plan of [17], we consider the task of predicting HIV infection using four different sets of immunologic markers: (1) all measured markers, (2) T cell markers, (3) antibody markers, and (4) no markers. In all analyses we also include the clinical covariates age, BMI, and behavior risk score unless otherwise specified. Set (2) includes T cell markers from [15]. Set (3) includes IgG, IgA, and IgG3 binding antibody markers, along with antibody Fc effector function markers [16, 17]. Set (1) is equal to the union of sets (2) and (3).

To evaluate prediction performance, we calculate the five-fold cross-validated area under the receiver operating characteristic curve (CV-AUC). As is common in many biomedical datasets with variables requiring resource-intensive laboratory measurement, the HVTN 505 immunologic marker dataset does not contain biomarker data for every participant from the full cohort. Instead, data are available from a subset of participants from a two-phase stratified sampling plan [4]. To account for this sampling design, the CV-AUC is computed using inverse sampling probability weighting (IPW), which are the inverse of the sampling probabilities determined by the two-phase sampling plan. Typically, all the cases are sampled because they are rare, thus their weights are 1. Only a small subset of controls is randomly sampled due to the abundance of controls, thus their weights are greater than 1. The weights for the HVTN 505 immunologic markers dataset are listed in Additional file 1: Table A.1. By incorporating the IPW in the CV-AUC calculation, prediction performance of a model based on two-phase samples can be generalized to the full cohort, and the formula [21] is defined as

$$\begin{aligned} \sum _{i \in D^{1}} \sum _{j \in D^{0}} w_i w_j I(P_i > P_j) / \sum _{i \in D^{1}} \sum _{j \in D^{0}} w_i w_j, \end{aligned}$$

where *i* and *j* are the case and control indexes, respectively; $D^1$ and $D^0$ are the case and control groups; *w* is the vector of IPW weights; and *P* is a prediction score, for RF models, it is the fraction of trees predicting cases. To obtain more stable CV-AUC estimates, we calculate the five-fold CV-AUC one-hundred times by using different random seeds to split the data and report the average CV-AUC over the one-hundred replications.

The performance of RF can suffer when there are too many input variables and when the numbers of cases and controls are imbalanced [18, 22]. Hence, in this section we consider the use of variable screening and class balancing to improve performance. Variable screening is a dimension-reduction technique often used in high-dimensional settings, and works by removing irrelevant variables and selecting informative variables. Screening algorithms can generally be classified into two categories: supervised and unsupervised screening [23]. The former screens variables based on the associations between input variables and an outcome; a representative example is penalized least squares or penalized likelihood. The latter considers only the input variables; well-known methods include clustering-based screening [24] and correlation-based screening [25]. In this paper, we employ lasso screening (a supervised method) that eliminates variables with zero coefficients estimated from lasso logistic regression models [26], which include the immunologic markers and the clinical covariates and employs five-fold cross-validation to select the lasso penalty.

Class imbalance occurs when one class has a much smaller number of observations than the other classes. In situations with class imbalance, most machine learning methods are biased toward the majority class (in the HVTN 505 example, the controls), and ignore the minority class; as a result, the performance of these methods can be unsatisfactory [18, 22]. Class balancing is a potential solution, wherein the class distribution is artificially rebalanced by randomly under-sampling the majority class or over-sampling the minority class. Many machine learning methods require the data to be pre-processed if class balancing is used; in contrast, RF can naturally incorporate class balancing since it can fit trees on class-balanced bootstrapped datasets that are obtained by modifying the sampling scheme when the ensemble is initialized. We study both under- and over-sampling separately to determine which results in the most improved performance in our setting.

To study the effects of variable screening, class balancing, and inverse sampling probability weighting on the performance of RF, we compare four RF models, each with and without variable screening (Table 1). The first is standard RF without class balancing or weighting, implemented using the *ranger* R package [27] with default settings. More detailed information on the default settings can be found in the Additional file 1: Section B. The second is RF with under-sampling, while the third is RF with over-sampling. In the five-fold cross-validation scheme, each training dataset has 20 cases and 100 controls. Thus, RF with under-sampling fits trees on bootstrap datasets with 20 cases and 20 controls, and RF with over-sampling fits trees on bootstrap datasets with 100 cases and 100 controls. These two methods are implemented using the *case.weights* and *sample.fraction* arguments in the *ranger* package. The former controls weights for bootstrap sampling, where observations with larger weights are more frequently represented in the bootstrap datasets, and the latter controls the fraction of observations to be sampled in the bootstrap datasets. Specifically, under-sampling is achieved by setting the argument *case.weights* equal to 5 for the cases and 1 for the controls, and setting the argument *sample.fraction* equal to 40/120. Some pre-processing is necessary for over-sampling, because the *ranger* package does not allow *sample.fraction* to be greater than 1. Here, we first create a training dataset that has 100 cases and 100 controls by randomly over-sampling the cases, and then fit a RF model on the modified training dataset by setting *case.weights* equal to 1 for all observations and *sample.fraction* equal to 200/200. The final model we consider is RF with IPW. This is implemented by setting *case.weights* equal to the IPW weights.

The results of this experiment are presented in Table 1. All RF models with screening outperform their counterparts without screening. This is most likely due to excessive overfitting when variable screening is not applied. The RF algorithm constructs individual trees with maximal depth without pruning [5]. Without screening, the RF algorithm will by chance use many noisy predictors in the tree construction and the resulting model will fail to generalize well. We examine this in more detail in Additional file 1: Section C.

The effects of under- and over-sampling depend on whether variable screening is applied. Without screening, class balancing methods confer substantial improvement over the standard RF. This is a well-known phenomenon [28] and can be attributed to the fact that RF models construct trees to minimize Gini impurity, which, unlike AUC, is sensitive to class prevalence [19, 29]. With screening, using class balancing methods leads to a slight decrease in performance in some sets of markers. That variable screening can counter class imbalance has been observed before [30, 31], though the reasons for this are not well understood. The decrease in prediction performance may be because under-sampling and over-sampling lead to degradation in data quality by throwing away data in the majority class and introducing duplicate data in the minority class, respectively.

The effects of using IPW also depend on variable screening. This is because on the one hand, using IPW makes the criterion function in the training step align more closely with the prediction performance metric; on the other hand, IPW may exacerbate the class imbalance problem in RF training. The results show that when variable screening is not applied, the RF with IPW performs worse than the standard RF in almost all sets of markers. This result makes sense because IPW gives more weights to the controls, which make the bootstrapped datasets even more imbalanced. When variable screening is applied, the overall impact of using IPW is reversed. With and without using IPW performs similarly for all markers and no markers; using IPW weighting performs slightly better for T cell markers and antibody markers.

**Table 1:** Comparison of CV-AUCs obtained by standard random forest (RF), random forest with under-sampling (RF_under), random forest with over-sampling (RF_over), and random forest with inverse sampling probability weights (RF_ipw), including results obtained without variable screening and results obtained with variable screening

	No screening				Screening			
	RF	RF_under	RF_over	RF_ipw	RF	RF_under	RF_over	RF_ipw
All markers	0.679	0.732	0.711	0.657	0.824	0.806	0.806	0.824
T cell markers	0.718	0.714	0.715	0.708	0.812	0.780	0.799	0.819
Antibody markers	0.605	0.656	0.628	0.579	0.708	0.722	0.696	0.711
No markers	0.442	0.452	0.448	0.443	0.442	0.452	0.448	0.443

Clinical covariates (age, BMI, and a risk behavior score) are always included

#### Hyperparameter tuning

The performance of RF may be further improved by tuning its hyperparameters. Although there are many hyperparameters in the RF algorithm, we explore three that have been shown to have the most impact on prediction performance [32]. These are (1) the number of variables randomly sampled as candidates at each split, (2) the minimum size of terminal nodes, and (3) the number of observations that are drawn for each tree. Note that tuning the size of terminal nodes is equivalent to tuning the depth of trees. We use the *tuneRanger* R package [33] to search over a grid of these hyperparameters for an optimal set of the hyperparameters based on out-of-bag AUC, which is AUC calculated on out-of-bag data that are not selected into the bootstrapped data in the initial stage of the RF algorithm (Fig. 1), through sequential model-based optimization. The optimal set is a set of hyperparameters that achieves the highest out-of-bag AUC among 50 to 100 sets of hyperparameters. More detailed information for the *tuneRanger* R package can be found in the Additional file 1: Section B.

To explore the effect of hyperparameter tuning, we compare the performance of standard RF and tuned RF. The standard RF is fit using the default hyperparameter values specified in the *ranger* R package, while the tuned RF is fit using the *tuneRanger* R package. For both methods, we use variable screening, but not class balancing or inverse sampling probability weighting. The design of the experiment is the same as before. Table 2 shows that tuning does not have a clear-cut effect on performance. When antibody markers alone or no markers are used, tuning increases performance; but when either all markers or T cell markers alone are used, tuning decreases performance. This is likely due to overfitting to the out-of-bag samples under small sample sizes, a phenomenon that has been observed in the econometrics literature [e.g. 34]. A similar phenomenon has also been observed in Kaggle competitions, where there are two testing datasets, a public leaderboard dataset and a larger private leaderboard dataset, and overfitting to the public testing dataset can decrease performance on the private testing dataset [35]. Another potential reason for explaining the observation may be that out-of-bag AUC, an optimization criterion in the tuning algorithm, does not consider IPW and would not align with the prediction performance metric CV-AUC, which incorporates IPW. It might select a sub-optimal set of hyperparameters, and tuning was not always successful. In Table 2 the CV-AUCs for both standard and tuned RF are below 0.5 when no markers are used. This is still true if we evaluate CV-AUC without using weights. One explanation for these results is overfitting to the training subset. For simplicity, suppose there is a single clinical covariate, and it shows no association with the outcome in a dataset. When the dataset is split into a training subset and a validation subset, by chance there may arise some association in the training subset. And because there is no overall association in the full dataset, there will also be association in the opposite direction in the validation subset, which will lead to CV-AUC less than 0.5.

**Table 2:** Comparison of CV-AUC of standard random forest (RF) and tuned random forest (tRF). Screening is applied to both methods, but not class balancing or inverse sampling probability weighting

	RF	tRF
All markers	0.824	0.807
T cell markers	0.812	0.802
Antibody markers	0.708	0.721
No markers	0.442	0.455

Clinical covariates (age, BMI, and a risk behavior score) are always included

#### Summary of approaches

Based on the results in Tables 1 and 2 , for the remainder of our analyses we will perform random forest model training with variable screening, without class balancing and without hyperparameter tuning. The choice of using IPW is more nuanced. Since using IPW leads to some improvement when variable screening is applied, we will use IPW in RF training.

### Stacking random forest and generalized linear models

When the outcome and the input variables have a simple linear relationship, it is possible that RF based on nonlinear modeling may be overly complex and ineffective. We see this in the case of clinical covariates only, where the CV-AUC of RF is 0.443; in contrast, a generalized linear model (GLM) has a CV-AUC of 0.624, where we use a logistic regression model as GLM. It suggests that the bias-variance trade-off associated with using RF may not always work in its favor in small-sample settings. To further examine this issue, we compare the performance of RF and GLM on each of the four sets of markers defined before, with and without the clinical covariates. Variable screening and inverse sampling probability weighting are applied for both methods, and we implement GLM using the *glm* R function with the *weights* argument for the weighting, which controls prior weights for subjects. The results, given in Table 3, show that the performance of GLM improves when the clinical covariates are added to each of the four sets of markers. The impact of adding clinical covariates on the performance of RF depends on the set of markers to be analyzed. For all markers and T cell markers, adding the clinical covariates results in improved performance; for antibody markers and no markers, adding the clinical covariates decreases performance. Furthermore, RF outperforms GLM for all markers and T cell markers when the clinical covariates are included, and GLM outperforms RF for antibody markers and no markers whether or not the clinical covariates are included. These results motivated us to consider an approach that incorporates different models and marker sets to improve prediction performance.

**Table 3:** Comparison of CV-AUCs of generalized linear models (GLM) and random forest (RF), with and without clinical covariates

	GLM		RF	
	No covariates	Covariates	No covariates	Covariates
All markers	0.810	0.813	0.808	0.824
T cell markers	0.781	0.793	0.806	0.819
Antibody markers	0.759	0.768	0.729	0.711
No markers	0.500^a^	0.624	0.500*	0.443

Screening is applied for both GLM and RF, but class balancing is not done for RF. Inverse sampling probability weights are used in both GLM and RF training

^a^Denotes theoretical values

Stacking [36] is an ensemble machine learning method that combines several different candidate learners into one meta-learner to improve prediction performance. The algorithm is composed of two steps: first, it trains several candidate learners and generates out-of-sample prediction scores, which are the estimated probabilities of being a case, by splitting the training data into a subset for fitting and a subset for making prediction scores; second, a meta-learner aggregates the out-of-sample prediction scores into a single prediction. Breiman [37] further developed the method by restricting to nonnegative weights when combining candidate learner prediction scores.

We propose using stacking to combine GLM learners and RF learners to further improve prediction performance. Based on the results in Table 3, we consider stacking GLM trained on antibody markers and clinical covariates and RF trained on T cell markers and clinical covariates. For comparison, we also examine three related stacking models by replacing antibody markers and/or T cell markers with all markers.

We implement stacking using the *caretEnsemble* R package [38], with ten-fold cross-validation [recommended by 37] for fitting candidate learners and obtaining out-of-sample prediction scores. To combine prediction scores from the different learners, we use logistic regression models with nonnegative coefficients. Finally, we use an outer loop of five-fold cross-validation to evaluate the performance of stacking and repeat the entire process one-hundred times as described before.

Table 4 shows the average CV-AUC of the four stacking models, along with the CV-AUCs of two RF models to facilitate comparison. The impact of stacking on the performance appears to depend on which candidate learners are used. In the top three rows, where the two stacking methods are based on RF trained on T cell markers and clinical covariates, the two stacking models show a relatively large improvement over RF, and the Pearson correlation coefficients between out-of-sample prediction scores from the RF model and those from the two GLM models are 0.26 and 0.65 (average over 100 replicates), respectively. In the bottom three rows, where the two stacking methods are based on the RF trained on all markers and clinical covariates, the performance of the two stacking models is rather close to the RF, and the Pearson correlation coefficients between out-of-sample prediction scores from the RF model and those from the two GLM models are 0.49 and 0.80 (average over 100 replicates), respectively. These observations are consistent with the well-known fact that stacking tends to be ineffective when the candidate learner prediction scores are similar to each other and the most effective stacking is achieved by combining dissimilar prediction scores [37].

For the best stacking model (RF: T cell markers + GLM: Antibody markers), the logistic regression model meta-learner combines the prediction scores from RF and GLM with coefficients 0.686 and 0.314 (average over 100 replicates), respectively. This suggests that there is more information in the T cell markers than in the antibody markers. The reason we stack together RF trained on the T cell markers and GLM trained on the antibody markers and not the other way around is because Table 3 suggests that RF works better than GLM on the T cell markers and GLM works better than RF on the antibody markers. Indeed, the stacking model RF: Antibody markers + GLM: T cell markers has an estimated CV-AUC of 0.797.

**Fig. 2:**
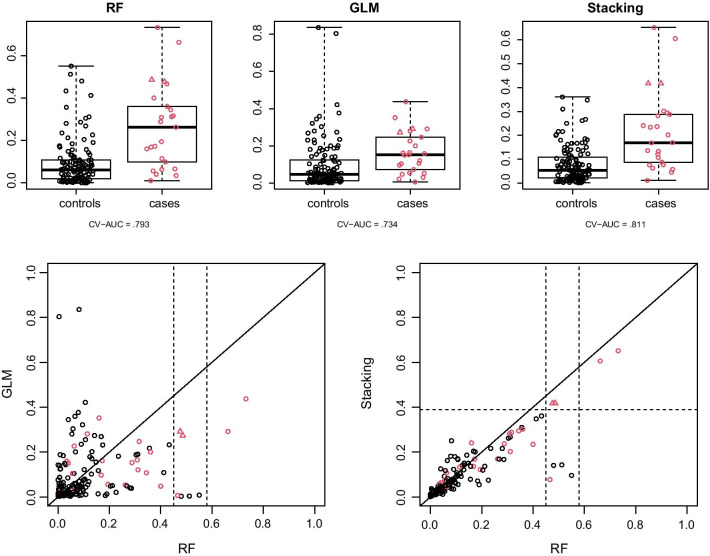
Top: boxplots of three prediction scores by cases and controls from one 5-fold cross validation. Bottom: scatterplots of these prediction scores. Cases are shown in red, and controls are shown in black. Study volunteers 180 and 183 are plotted as triangles

To help elucidate how stacking helps improve prediction performance, we examine the prediction scores from RF, GLM, and the stacking model for one 5-fold cross-validation replicate. The top row of Fig. 2 shows three boxplots of prediction scores by cases and controls. We focus on two cases (study volunteers 180 and 183) that are plotted with triangle plotting symbols. Neither RF nor GLM prediction scores set them apart from all the controls, but their stacking prediction scores are higher than all the controls. The bottom-left panel of Fig. 2 shows a scatterplot of the RF and GLM prediction scores. There are six samples between the two vertical dashed lines, including three cases and three controls. All six samples have high RF prediction scores, but only study volunteers 180 and 183 have high GLM prediction scores. The bottom-right panel of Fig. 2 shows a scatterplot of the RF and stacking prediction scores. The stacking prediction scores of study volunteers 180 and 183 are higher than all the controls. Since subjects with high RF prediction scores have low levels of T cell markers and subjects with high GLM prediction scores have low levels of antibody markers, these results suggest that if a subject has both low levels of T cell markers and low levels of antibody markers, they are more likely to be infected with HIV.

**Table 4:** Comparison of CV-AUCs of four stacking models and two random forest models

	CV-AUC
RF: T cell markers + GLM: antibody markers	0.838
RF: T cell markers + GLM: All markers	0.831
RF: T cell markers	0.819
RF: All markers + GLM: ntibody markers	0.821
RF: All markers + GLM: All markers	0.821
RF: All markers	0.824

Screening is applied for both GLM and RF, but class balancing is not done for RF. Inverse sampling probability weights are used in both GLM and RF training. Clinical covariates are included in the predictors of all RF and GLM models

## Conclusion

In this paper we studied the optimal use of random forest (RF) for classification on a dataset from a two-phase sampling design, a common situation in prevention studies of public health importance, which often have a small number of disease endpoints. We considered the HVTN 505 phase III HIV vaccine efficacy trial dataset, which contains 25 cases and hundreds of immunologic markers.

First, we found that variable screening before applying RF substantially improves RF prediction performance, as measured by weighted CV-AUC. This improvement is likely a result of avoiding overfitting. Second, while class balancing improves RF prediction when variable screening is not applied, it has a negative impact on performance when variable screening is applied. Third, the impact of inverse sampling probability weighting (IPW) similarly depends on whether variable screening is applied. Without variable screening, IPW led to poorer performance due to the class imbalance problem in the RF training step. Relatively more weighting to the majority class causes bootstrapped samples to be even more imbalanced, resulting in trees with poor prediction performance for the minority class. However, with variable screening, IPW actually improved performance for almost all subsets of markers. Inverse sampling probability weighting almost always leads to better results for GLM, regardless of whether variable screening is applied (Additional file 1: Table A.2). Fourth, we investigated the impact of hyperparameter tuning on the performance of RF. Tuning was not always successful, possibly due to overfitting to the out-of-bag data under small sample sizes. Lastly, we found that RF under-performed simple linear methods such as GLM for some marker sets, and the use of stacking to combine RF and GLM models achieved improved prediction performance. The performances of the stacking models were tied to the similarities between candidate learner prediction scores. The best performance came from stacking a random forest model trained on the T cell markers and the clinical covariates and a GLM trained on the antibody markers and the clinical covariates, and their Pearson correlation coefficient was 0.26, the lowest among the four stacking models we tried.

The differences in CV-AUC between the best stacking model and the other models in Table 4 range between 0.007 and 0.019. Differences of this magnitude can be clinically meaningful [e.g. 39, 40]. One way to assess the variability of these differences is to examine their distributions across the 100 replicates of 5-fold cross validation and perform Wilcoxon signed rank tests. All the p-values from the tests are highly significant at $<0.001$, suggesting that the performance of the best stacking model does not depend on a specific random split of the data. Evaluating the variability of the CV-AUC on the population level is a more challenging problem, e.g., there is no known theoretical results that ensure the success of the Efron bootstrap [41] procedure for CV-AUC, and will be an interesting future research direction.

## Supplementary Information

**Additional file 1**: supplement.pdf contains additional study results regarding hyperparameters tuning, variable screening, and two-phase studies.

## Data Availability

All the source data and code are available at https://atlas.scharp.org/cpas/project/HVTN%20Public%20Data/HVTN%20505/begin.view and https://github.com/shan-stat/rf_hvtn505

## Abbreviations

HVTN: HIV vaccine trials network
RF: Random forests
BMI: Body Mass Index
GLM: Generalized linear models
CART: Classification and regression trees
CV-AUC: Cross-validated area under the receiver operating characteristic curve
IPW: Inverse sampling probability weights
