# Supplementary material for: Improving random forest predictions in small datasets from two-phase sampling designs
Source: BMC Med Inform Decis Mak. 2021 Nov 22;21:322. doi: 10.1186/s12911-021-01688-3 (PMC8607560; doi:10.1186/s12911-021-01688-3)
Supplement: Supplementary file 1 — Additional file 1: supplement.pdf contains additional study results regarding hyperparameters tuning, variable screening, and two-phase studies. [file 12911_2021_1688_MOESM1_ESM.pdf]

Supplementary Materials for Improving Random Forest Predictions in Small Datasets from Two-phase Sampling Designs

Sunwoo Han, Brian D. Williamson, Youyi Fong

A Additional tables and figures

	Case #	Control		
		# Sampled	# Available	IPW
White & $18.4 \leq$ BMI < 25.0	6	30	91	3.033
White & $25.0 \leq$ BMI < 29.8	5	25	56	2.240
White & $29.8 \leq$ BMI < 40.0	4	20	42	2.100
Black_Hispanic & $18.4 \leq$ BMI < 25.0	6	26	27	1.038
Black_Hispanic & $25.0 \leq$ BMI < 29.8	3	13	16	1.231
Black_Hispanic & $29.8 \leq$ BMI < 40.0	1	11	18	1.636

Table A.1: Detailed information on the two-phase sampling design of the HVTN 505 immunologic biomarkers study. There were six demographics strata. All available cases are sampled and their inverse sampling probability weights (IPW) are all 1.

	No screening		Screening	
	GLM	GLM_ipw	GLM	GLM_ipw
All markers	0.489	0.489	0.804	0.813
T cell markers	0.496	0.496	0.784	0.793
Antibody markers	0.511	0.510	0.770	0.768
No markers	0.621	0.624	0.621	0.624

Table A.2: Comparison of CV-AUC of GLM with and without inverse sampling probability weights.

B Additional information for hyperparameters in RF

Standard RF is fitted with the *ranger* function from the *ranger* R package (Wright and Ziegler, 2017). The key parameters to the function and their default values are listed below:

- *mtry*: the number of variables randomly sampled at each split. The default is square root of the number of variables (rounded down to the nearest integer).
- *min.node.size*: the minimum size of terminal nodes. The default is 1 for classification problem, where trees are grown to the maximum possible. Setting larger values causes smaller trees to be grown, also known as pruning.
- *sample.fraction*: fraction of observations that are drawn for each tree during bootstrapping. The default is 1, indicating no subsampling.
- *case.weights*: weights for bootstrap sampling. The default is NULL, in which case the bootstrap is not weighted.
- *num.trees*: the number of trees. The default is 500.

The tuning algorithm is performed with the *tuneRanger* function from the *tuneRanger* R package (Probst et al., 2019). The major parameters to the function and their default values are listed below:

- *measure*: performance measure to optimize hyperparameters. The default is brier score for classification, but AUC is used in this study.
- *iters.warmup*: the minimum number of searches to find an optimal hyperparameter. The default is 30, but 50 is used in this study.
- *iters*: the maximum number of searches to find an optimal hyperparameter. The default is 70, but 100 is used in this study.
- *tune.parameters*: hyperparameters. The default is *mtry*, *sample.fraction*, and *min.node.size*. Each hyperparameter is tuned over the ranges as follows: (1) *mtry*: $[0, p]$ with p being the number of predictors; (2) *sample.fraction*: $[0.2n, 0.9n]$ with n being the number of observations; (3) *min.node.size*: $[(0.2n)^x]$ with x being from 0 to 1.

We do not consider the number of trees (*num.trees*) in the hyperparameter tuning because more trees produce more stable estimates in general. The 500 trees by default may suffice to obtain a stable estimate in the HVTN 505 dataset with small sample sizes. In the literature, empirical results with many datasets showed that constructing the first 100 trees makes the biggest performance gain (Oshiro et al., 2012; Probst and Boulesteix, 2017).

C Comparison of random forest models with and without variable screening

We compared three sets of prediction performance between RF without screening and RF with screening using all immunologic markers: (1) AUC on the bootstrapped data, i.e. in-bag data, which is used to construct trees in RF, (2) AUC on the training data (the training data includes observations sampled into the bootstrapped data and observations left out of the bootstrapped data), and (3) CV-AUC, which is the AUC on the validation data. For (3), the results are the same as those in Table 1 in the manuscript. For (1) and (2), we averaged the AUCs from 100 replicates of 5-fold cross-validation schemes just as for (3). The results are shown in Table C.1.

	RF without screening	RF with screening
AUC on the bootstrapped data	1.000	0.999
AUC on the training data	0.831	0.854
AUC on the validation data (CV-AUC)	0.679	0.824

Table C.1: Three AUCs for RF with and without screening using all immunologic marekrs.

First, the results show that without screening the AUC on the bootstrapped data is all 1. This is because the RF algorithm constructs individual trees with maximal depth without pruning (Breiman, 2001). When there are a lot of features to choose from, all the terminal nodes are pure (all cases or all controls, the right panel in Figure C.2), and the resulting RF model has perfect prediction performance on the bootstrapped data. With screening, the number of features is limited, and the RF algorithm sometimes (3.4% of all trees) has difficulty producing all pure terminal nodes (the left panel in Figure C.2) and so the AUC is close to but not 1. This provides the first indication that RF without screening has a bigger overfitting problem than RF with screening.

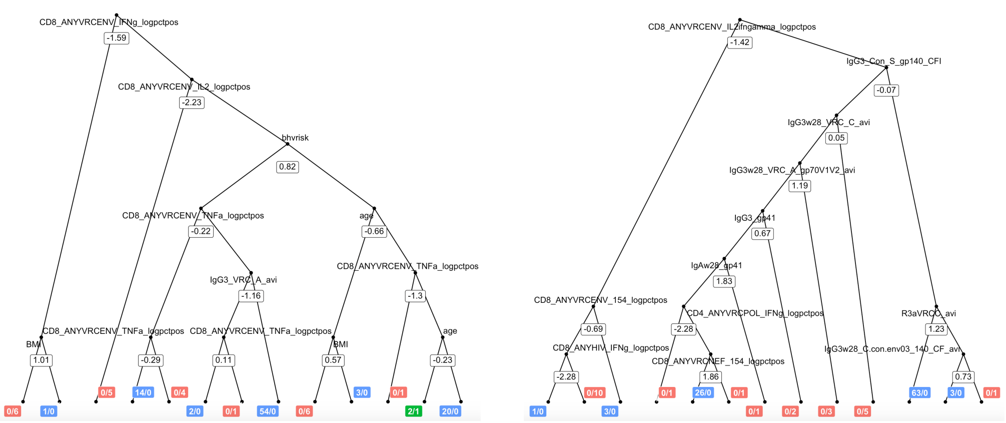

Figure C.2: A tree in RF with screening (left) and without screening (right). The split predictor and value are displayed at each split. The terminal nodes contain information about the number of controls and cases (control/case), where red and blue are pure (containing cases only or controls only, respectively) and green is impure.

Second, RF with screening has a bigger AUC on the training data than RF without screening. The training data differs from the bootstrapped data in that it contains the out-of-bag data that are not sampled into the bootstrapped data. This suggests the RF model with screening generalizes better than the RF model without screening. Third, RF with screening has a far bigger AUC on the validation data than RF without screening. These results are similar to the second set of results, but the over-fitting of RF without screening is more pronounced here because none of the validation data are used in model training.

The overfitting is fundamentally caused by the fact that the RF algorithm can achieve near perfect separation using any predictors. Without screening, there are many noise predictors, and we expect RF to use many noise predictors in tree construction, which would lead to poor generalization performance. Figure C.3 shows the number of times each predictor is used for splitting in RF in the experiments described above. RF with screening uses only eight predictors that pass screening. In contrast, RF without screening uses almost all the predictors as we expect (the first eight predictors are shown in blue, and they are the same ones used by RF with screening).

All together, these results suggest that screening helps reduce overfitting by only using informative predictors in tree construction.

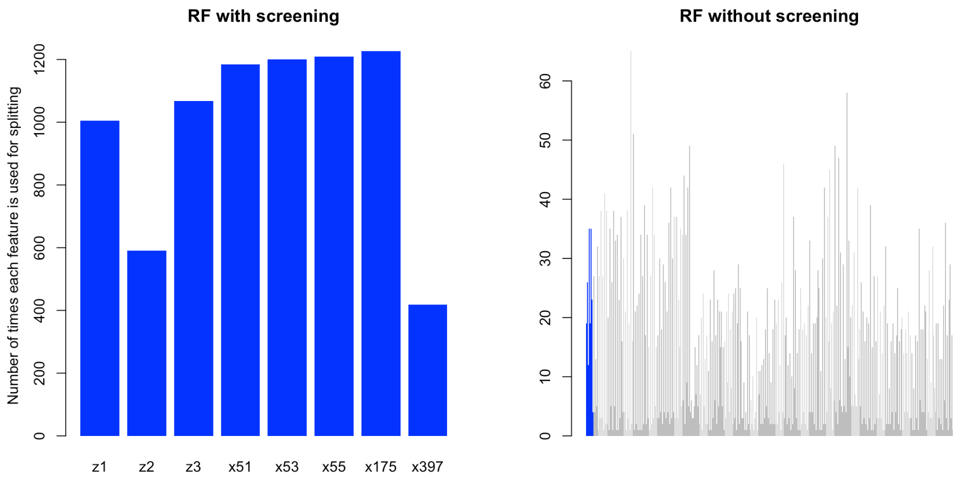

Figure C.3: The number of times each predictor is used for splitting in RF with screening (left) without screening (right). In the right panel, the first eight predictors, colored blue, are the same predictors shown in the left panel.

D Two-phase studies

Two-phase studies are useful when we have a large study (phase 1) in which it is only practical to measure some predictor variables of interest for a subset of study participants (phase 2). More formally, in phase 1 we draw a cohort sample that is representative of the target population relevant to the clinical application at hand. Every participant's disease status and easy to measure covariates are obtained. In phase 2, a subsample is drawn without replacement from the phase 1 sample. The probability of a phase 1 individual being drawn into phase 2 can depend on certain stratification variables, including disease status. There are different types of two-phase study designs, including case-control (Breslow, 1996) and case-cohort (Prentice, 1986; Borgan et al., 2000) studies. So long as each phase 1 participant's sampling probability is known, the bias in sampling can be corrected through inverse probability weighting or other techniques.

Let's illustrate two-phase studies with the example used in Breslow et al. (2009). The phase 1 sample is a cohort of 12,345 study participants of the Atherosclerosis Risk in Communities (ARIC) study who were free from coronary heart disease, had plasma samples taken at their second follow-up visit, and had no missing values of phase 1 covariates. The biomarkers Lp-PLA 2 and C-reactive protein could not be measured for every participant in the study. A phase 2 sample was taken stratified by race, gender, and age as well as case status for a total of k=9 strata. The table below lists the number of phase 1 and phase 2 participants in each stratum. A weight is computed for each stratum and equals the number of phase 1 participants divided by the number of phase 2 participants in the stratum.

Breslow et al. (2009) **Table 1.** Stratified Sampling Design for the Atherosclerosis Risk in Communities Study

	Non-CHD Cases (Controls)								CHD Cases	Totals, no.
	Black				White					
	Female		Male		Female		Male			
	Age <55 Years	Age ≥55 Years	Age <55 Years	Age ≥55 Years	Age <55 Years	Age ≥55 Years	Age <55 Years	Age ≥55 Years		
Stratum (k)	1	2	3	4	5	6	7	8	9	
Cohort N_k	1,133	719	598	393	2,782	2,213	1,959	1,818	730	12,345
Sample n_k	59	54	42	71	88	154	117	147	604	1,336
Weights N_k/n_k	19.2	13.3	14.2	5.5	31.6	14.4	16.7	12.4	1.2	

Abbreviation: CHD, coronary heart disease.

References

Borgan, O., Langholz, B., Samuelsen, S.O., Goldstein, L. and Pogoda, J. (2000), "Exposure stratified case-cohort designs," *Lifetime data analysis*, 6, 39–58.

Breiman, L. (2001), "Random forests," *Machine learning*, 45, 5–32.

Breslow, N.E. (1996), "Statistics in epidemiology: the case-control study," *Journal of the American Statistical Association*, 91, 14–28.

Breslow, N.E., Lumley, T., Ballantyne, C.M., Chambless, L.E. and Kulich, M. (2009), "Using the whole cohort in the analysis of case-cohort data," *American Journal of Epidemiology*, 169, 1398–1405.

Oshiro, T.M., Perez, P.S. and Baranauskas, J.A. (2012), "How many trees in a random forest?" in *International workshop on machine learning and data mining in pattern recognition*, pp. 154–168, Springer.

Prentice, R.L. (1986), "A case-cohort design for epidemiologic cohort studies and disease prevention trials," *Biometrika*, 73, 1–11.

Probst, P. and Boulesteix, A.L. (2017), "To tune or not to tune the number of trees in random forest." *J. Mach. Learn. Res.*, 18, 6673–6690.

Probst, P., Wright, M.N. and Boulesteix, A.L. (2019), "Hyperparameters and tuning strategies for random forest," *Wiley Interdisciplinary Reviews: Data Mining and Knowledge Discovery*, 9, e1301.

Wright, M.N. and Ziegler, A. (2017), "ranger: A Fast Implementation of Random Forests for High Dimensional Data in C++ and R," *Journal of Statistical Software*, 77, 1–17.
